# Tissue-based molecular markers in upper tract urothelial carcinoma and their prognostic implications

**DOI:** 10.1590/S1677-5538.IBJU.2017.0204

**Published:** 2018

**Authors:** Ricardo L. Favaretto, Stênio C. Zequi, Renato A. R. Oliveira, Thiago Santana, Walter H. Costa, Isabela W. Cunha, Gustavo C. Guimarães

**Affiliations:** 1 Departamento de Cirurgia Pélvica, Serviço de Urologia AC Camargo Cancer Center, São Paulo, Brasil, São Paulo, Brasil; 2 Departamento de Patologia, AC Camargo Cancer Center, São Paulo, Brasil

**Keywords:** Carcinoma, Biomarkers, Prognosis

## Abstract

Upper tract urothelial carcinoma (UTUC) is a rare and aggressive disease that is associated with high rates of recurrence and death. Radical nephroureterectomy (RNU) with excision of the bladder cuff is considered the standard of care for high-risk UTUC, whereas kidney-sparing techniques can be indicated for select patients with low-risk disease. There is a significant lack of clinical and pathological prognostic factors for stratifying patients with regard to making treatment decisions. Incorporation of tissue-based molecular markers into prognostic tools could help accurately stratify patients for clinical decision-making in this heterogeneous disease. Although the number of studies on tissue-based markers in UTUC has risen dramatically in the past several years—many of which are based on single centers and small cohorts, with a low level of evidence—many discrepancies remain between their results. Nevertheless, certain biomarkers are promising tools, necessitating prospective multi-institution studies to validate their function.

## INTRODUCTION

Upper tract urothelial carcinoma (UTUC) is a rare disease, accounting for approximately 5% to 10% of all urothelial malignancies, and has an estimated annual incidence of 2 per 100.000 inhabitants in Western countries (1). Although improvements in imaging and endoscopic techniques have led to stage migration in UTUC, it remains an aggressive malignancy with high recurrence and progression rates.

Radical nephron-ureterectomy with excision of the bladder cuff (RNU) is the standard treatment for UTUC (1, 2). However, kidney-sparing techniques can be indicated for select patients with low-risk UTUC, whereas multimodal therapies, such as perioperative chemotherapy and extended lymph node dissection, in addition to RNU, are considered for high-risk disease (1, 2).

The correct preoperative identification of patients who could benefit from these procedures is complicated. In the past two decades, many studies have examined the clinical, operative, and pathological prognostic factors in UTUC (2). Consequently, several predictive and prognostic tools, based on various statistical techniques, have been developed to provide accurate estimates of outcomes (3-5).

Yet, the predictive accuracy of these prognostic models remains low. Thus, guiding patients and physicians in selecting the best treatment options and follow-up strategies in UTUC is challenging, necessitating better approaches to counseling individual patients. Recent studies on prognostic biomarkers in urothelial carcinoma are promising and might benefit outcomes by improving risk stratification and decision-making (6-8).

Molecular markers are molecules that are produced by a tumor and are detectable and quantifiable in specimens, such as tissue, blood, and urine. Ideal biomarkers improve the accuracy when integrated into prognostic models, allowing one to counsel patients regarding treatment decisions (9). Biomarkers have been studied extensively in urothelial carcinoma of the bladder (UCB), several of which are established independent prognostic factors that are associated with features of biologically and clinically aggressive UCB (10-12). However, the carcinogenetic mechanisms in UTUC differ from those of urothelial carcinoma of the lower urinary tract; thus, the molecular alterations that occur in one setting might not be able to be extrapolated to the other (8).

In the past several years, many collaborative groups have published studies on molecular markers as prognostic factors in UTUC, especially tissue-based markers (6-8). However, due to the low incidence of the disease, no randomized controlled trial has been reported. Further, few reviews have discussed this issue, and none has focused on tissue-based markers.

The objective of this review is to detail recent data on tissue-based markers in UTUC, how each of these markers functions in oncogenesis, their prognostic impact, and how they influence clinical decision-making in this disease.

### Review Criteria

A nonsystematic literature search was conducted using the PubMed/Medline database to identify English articles (original, review, and editorial articles) on tissue-based molecular markers in UTUC using the following key words and phrases: “upper urinary tract carcinoma” or “upper tract transitional cell carcinoma” or “upper tract urothelial carcinoma” AND “molecular markers” or “biomarkers.” To select relevant articles for the different sections of the manuscript, additional references were collected by cross-referencing the bibliography of the selected articles. We chose articles that were published between 1995 and 2016, because the data on molecular markers in UTUC have become available only recently. Older articles were considered only if they were pertinent. The most relevant articles were examined and selected, based on the consensus of all authors.

### Tissue-based markers in UTUC

Recent data by the Cancer Genome Atlas (TCGA) project have changed the landscape of urothelial carcinoma research (13), performing a comprehensive genetic and molecular analysis that has provided novel insights into the molecular pathways that underlie carcinogenesis, disease progression, and possible therapeutic targets. Some of the molecular markers that mediate tumorigenesis can be measured and correlated with oncological outcomes. In UTUC, most studies on molecular markers are related to tissue-based markers (Table-1 and Table-2). Many of the regulatory proteins that are involved in cell cycle regulation, cell growth, proliferation and differentiation, signal transduction, angiogenesis, apoptosis, and cell adhesion have been recently studied in UTUC. The major limitations of these studies are their retrospective nature and their small sample size, due primarily to the low incidence of the disease.

**Table 1:** Tissue-based markers in upper tract urothelial carcinoma and their molecular functions.

TISSUE-BASED MARKERS		FUNCTION
**Cell cycle regulation**		
	p53	Tumor suppressor gene that maintains genomic stability by preventing genomic mutation. When mutated, tumor suppression is compromised, and tumors can develop.
	Cyclins	Proteins that regulate progression through the cell cycle (G1/S transition) by activation of cyclin-dependent kinase. Dysregulation of CDK-cyclin complexes can promote tumor cell growth.
	p21 and p27	Members of the cyclin-dependent kinase family. Function as tumor suppressors, and loss of their expression is associated with tumor development and progression.
**Cell growth, proliferation, and signal transduction**		
	Ki67	Nuclear protein associated with cellular proliferation. Marker of growth factor for a specific cell population.
	HER-2	Member of the ErbB family of receptors (plasma membrane-bound receptor tyrosine kinases). Plays an important role in tumor cell growth and development. Amplification or overexpression of HER-2 stimulates PI3K/AKT pathway and is associated with the development and progression of cancer.
	EGFR	Transmembrane glycoprotein with tyrosine kinase activity and also a member of the ErbB family of receptors. EGFR activation stimulates intrinsic intracellular protein-tyrosine kinase activity, leading to DNA synthesis and cell proliferation. EGFR overexpression or upregulation is associated with malignant tumors.
	NF-κB	Complex protein that regulates genes which controls cell proliferation and survival. NF-κB is inactive and sequestered in the cytoplasm. Several stimuli can activate and release NF-κB, which translocates to the nucleus. In the active form, it can regulate the expression of several genes associated with anti-apoptosis, inflammation, proliferation, invasion, and angiogenesis.
**Angiogenesis**		
	HIF-1α	Transcription factor that plays an important role in oxygen homeostasis. Levels of H**I**F-1 α subunit can increase rapidly in response to hypoxia and upregulate a number of factors important for tumor expansion.
	Vasohibin-1	Angiogenic molecule expressed in endothelial cells and upregulated by VEGF and fibroblast growth factor-2. VASH1 expression is restricted to endothelial cells of blood vessels in the tumor stroma and has a positive correlation with MVD.
	Thrombospondin-1	Potent inhibitor of angiogenesis in physiological and pathological conditions and inhibits proliferation of tube-like structures in endothelial cells. TSP-1 expression is inversely related to MVD.
	FGFR3	Part of a tyrosine kinase receptor family that regulates several cellular processes, such as growth, differentiation, and angiogenesis.
**Cell differentiation**		
	Uroplakin	Specific differentiation product of urothelial cells and is undetectable in nonurothelial tissues. Present at the apical surface and cytoplasm of umbrella cells and can be used as marker for urothelial differentiation.
**Cell adhesion/EMT**		
	Snail	Considered an EMT transcription factor that can repress E-cadherin and therefore regulate tumor progression and metastasis.
	E-cadherin	Calcium-dependent glycoprotein essential to epithelial tissue integrity. Expressed in epithelial cells, where cytoplasmic domain binds with β or γ subtype of catenin proteins and secures attachment to the actin microfilament, thus ensuring cytoskeletal integrity and stable cellular adhesion. Decreased E-cadherin can lead to loss of cellular adhesion, resulting in invasive tumors.
	N-cadherin	Expressed by mesenchymal cells. Decreased expression of E-cadherin and increase of N-cadherin have been established as features of EMT in epithelial malignancies and have been shown to be associated with the acquisition of aggressive phenotypes.
	Catenins	Family of proteins found in complexes with cadherins. They connect cadherins to actin filaments, ensuring cytoskeletal integrity and stable cellular adhesion.
	MMPs	Part of a family of proteases capable of degrading all kinds of extracellular matrix proteins. Tumor cell invasion and metastasis are biologically dependent on the proteolytic destruction of surrounding matrix components.
**Apoptosis**		
	Survivin	Member of a family of proteins that inhibit apoptosis by blocking downstream caspase activity.
	Bcl-2	Bcl-2 is an antiapoptotic protein that blocks caspase activation by inhibiting the mitochondrial release of cytochrome-c.
	Caspase	Proteases that can be activated by intrinsic or extrinsic pathways that induce cell death by apoptosis.

**Abbreviations:** CDK = cyclin-dependent kinase; EGFR = endothelial growth factor receptor; NF-κB = nuclear factor-κB; HIF-1α = hypoxia-inducible factor 1α; VEGF = vascular endothelial growth factor; MVD = microvessel density; VASH1 = vasohibin-1; TSP-1 = thrombospondin-1; FGFR3 = fibroblast growth factor receptor 3; MMPs = metalloproteinases; Bcl-2 = B-cell lymphoma 2 oncogene.

**Table 2:** Tissue-based markers in upper tract urothelial carcinoma and their prognostic implications.

TISSUE-BASED MARKERS		PATHOLOGICAL	BLADDER RECURRENCE	RFS	CSS	OS	REF.
**Cell cycle regulation**							
	p53	High pT stage; high grade		multivariate	multivariate	multivariate	14,15,16
	Cyclins			univariate			18,19
	p21						22
	p27	High pT stage; high grade				multivariate	20,22
**Cell growth, proliferation and signal transduction**							
	Ki67	High pT stage; high grade; LVI; sessile tumor; tumor necrosis, Cis, LN+	multivariate	multivariate	multivariate		23,24,25,26,27,28
	HER-2	High pT stage; high grade; LVI; tumor necrosis; LN+	multivariate	multivariate	multivariate	multivariate	30,31,32,33
	EGFR	High pT stage; high grade	multivariate	univariate			27,36
	NF-κB				multivariate	multivariate	38
***Angiogenesis***							
HIF-1α		High grade; sessile tumor		multivariate		multivariate	40
Vasohibin-1		High pT stage; high grade		multivariate	multivariate		42
Thrombospondin-1		High pT stage; high grade		univariate	univariate		46
FGFR3		Low pTstage; low grade					49,50
**Cell differentiation**							
Uroplakin		High pT stage; high grade			multivariate		52,53
**Cell adhesion/EMT**							
Snail		High pT stage; high grade; LVI		multivariate	multivariate		56
E-cadherin		High pT stage; high grade; LVI, tumor necrosis; Cis; LN+; sessile tumor; multifocality		univariate	univariate		58,59,60
N-cadherin		High pT stage; LN+; sessile tumor	multivariate	multivariate			59,61
Catenins		Larger tumors (>3cm)		multivariate	multivariate		63,64
MMPs		High pT stage; high grade; LVI; LN+		multivariate	multivariate	univariate	68,69,71
**Apoptosis**							
Survivin		High pT stage; LN+; LVI; tumor necrosis; sessile tumor		multivariate	multivariate		74,75
Bcl-2					multivariate		79
Caspase		High pT stage; high grade		univariate	univariate		74

**Abbreviations:** RFS = recurrence-free survival; CSS = cancer-specific survival; OS = overall survival; LVI = lympho-vascular invasion; Cis = carcinoma in situ; LN+ = lymph node metastasis; EMT = epithelial-mesenchymal transition; MMPs = metalloproteinases; EGFR = endothelial growth factor receptor; NF-κB = nuclear factor-κB; HIF-1α = hypoxia-inducible factor 1α; FGFR3 = fibroblast growth factor receptor 3; MMPs = metalloproteinases; Bcl-2 = B-cell lymphoma 2 oncogene; REF = references.

### Cell cycle regulation

#### p53

p53 is a tumor suppressor gene in chromosome 17p13 that preserves genomic stability by preventing genomic mutation. When p53 becomes damaged, tumor suppression is severely compromised, potentiating the formation of tumors.

p53 is one of the most widely investigated molecular markers in urothelial carcinoma, particularly with regard to its expression and function in the outcomes of patients with UTUC. Most studies agree that p53 expression is associated with features of aggressive disease, such as higher tumor grade and stage. However, its independent prognostic value is unknown when adjusted for other clinical and pathological characteristics by multivariate analysis. A systematic review and meta-analysis was recently published on the function of p53 in the survival of UTUC patients (14). Seven articles met the eligibility criteria for this review, which included 514 patients, finding that overexpression of p53 correlated with statistically significant differences in disease-free (DFS), cancer-specific (CSS), and overall survival (OS), suggesting that p53 is an independent prognostic factor in UTUC. Rey et al. were one of the first groups to examine the prognostic value of p53 and reported that its overexpression was significantly associated with tumor aggressiveness and patient survival in a small cohort of 83 patients (15).

In contrast, a recent review by Mitchell et al. analyzed 24 papers, in 5 of which multivariate analysis demonstrated that p53 expression had independent prognostic significance in UTUC, all of which contained potential statistical bias. The authors concluded that existing data do not support p53 as an independent prognostic marker in UTUC, requiring more prospective collaborative studies (16).

### Cyclins

Cyclins are a family of proteins that regulate progression through the cell cycle by activation of cyclin-dependent kinase (CDK). G1/S cyclins, which include cyclin A, cyclin D, and cyclin E, are essential for controlling the cell cycle during the G1/S transition. Cyclin levels usually modulate during the cell cycle and are strictly regulated. Overexpression of cyclin A and E and dysregulation of CDK-cyclin complexes can promote tumor cell growth and mediate the pathogenesis of cancer. In addition, high expression of cyclins A and E is associated with a poor prognosis in several types of cancer (17).

In UTUC, Liang et al. studied 340 patients with localized disease and found that expression of cyclin A was associated with worse metastasis-free survival (MFS) in the univariate but not multivariate analysis (18). Bogdanovic et al. retrospectively analyzed cyclin E levels in 24 patients and found no statistically significant association with tumor stage or grade (19).

### p21 and p27

p21 and p27 are members of the cyclin-dependent kinase family, functioning as tumors suppressors. The loss of their expression is linked to tumor development and progression in many malignancies.

In UTUC, Nakanishi et al. found that p27 expression declines significantly with increasing stage and grade, but no correlation was observed with the prognosis (20). In 2005, Fromont et al. analyzed a panel of molecular markers and failed to note any prognostic value of p27 expression (21). Recently, Sarsik and colleagues studied the immune-expression of p21 and p27 and correlated them with various clinical and pathological variables and OS (22). They reported that p21 and p27 are expressed in 52.9% and 88.2% of cases, respectively. Although no correlation was seen between loss of or lower p21 expression and clinico-pathological variables, they were associated with a shorter OS in the univariate and multivariate analysis. No noninvasive tumors showed a loss of p27, whereas one-third of invasive tumors did. This group concluded that additional research is needed to determine the function of p27 in UTUC, due to their small cohort (n=34) and the preliminary nature of their results.

### Cell growth and proliferation and signal transduction

#### Ki-67

Ki-67 is a nuclear protein that is associated with cell proliferation and is an excellent marker for determining growth factor for a specific cell population, with prognostic value for carcinomas of the prostate, breast, and brain.

The prognostic value of Ki67 in UTUC has been studied in the past several years. A Japanese group retrospectively analyzed 69 patients who were diagnosed with UTUC and determined that overexpression of Ki67 by immunohistochemistry correlated significantly with stage, grade, and lymphatic and vascular invasion (23). In survival analyses, Ki67 expression was a significant prognostic factor by univariate analysis but did not impact survival in the multivariate analysis. In 2005, a multicenter study examined the prognostic value of Ki67 in UTUC and found an association with poor prognosis and disease recurrence only in the univariate analysis (21).

In contrast, a recent study on specimens from 107 patients who underwent RNU found that Ki67 overexpression was linked to pathological stage and grade (24). In survival analyses, overexpression of Ki67 was an independent predictor of DFS and CSS. These results were posteriorly confirmed in a prospective study of 101 patients (25). Notably, Joung et al. attempted to identify immune-histochemical predictors that were associated with bladder recurrence in patients with UTUC who were subjected to RNU and found that Ki67 overexpression significantly predicts bladder cancer recurrence (26). These results were confirmed by Long et al. in a cohort of 320 patients (27). Thus, Ki67 can be used as a marker of bladder recurrence after RNU, anticipating intravesical therapies.

In 2015, the International Upper Tract Urothelial Carcinoma Collaboration published a multi-institution validation study of the predictive value of Ki67 in patients with UTUC (28). Immuno-histochemical staining for Ki67 was performed on a tissue microarray that was constructed from 475 patients who underwent radical surgery. Overexpression of Ki67 was significantly associated with higher pT stage, lympho-vascular invasion, sessile tumor architecture, tumor necrosis, concomitant carcinoma in situ, and regional lymph node metastasis. By multivariate analysis, Ki67 was an independent predictor of DFS and CSS. This group also observed a statistically significant improvement in Harrell’s C-index when Ki67 was included in their preoperative and postoperative prediction models for DFS and CSS, concluding that they validated Ki67 as an independent predictor of outcomes in UTUC patients.

#### HER-2

HER-2 (human epidermal growth factor receptor 2), also known as ErbB2, is a member of the ErbB family of receptors, which comprises four plasma membrane-bound receptor tyrosine kinases. HER-2 has important functions in tumor cell growth and development. Amplification or overexpression of HER-2 stimulates the phophoinositide-3 kinase (PI3K)/AKT pathway and is associated with the development and progression of certain aggressive types of cancer. In breast cancer, HER-2 amplification is found in nearly 25% to 30% of tumors and correlates with disease recurrence and poor survival (29). Targeting HER-2 with monoclonal antibodies in HER-2-overexpressing breast carcinomas improves survival and has become a standard treatment option (29).

Studies in urological tumors have shown that HER-2 overexpression in patients with UTUC who undergo radical therapy is rare. Vershasselt-Crinquette et al. measured HER-2 expression in a cohort of 83 patients (30) by immune-histochemical staining and in situ hybridization, observing overexpression and amplification was in 16% and 7% of subjects, respectively. However, patients with overexpression and amplification of HER-2 were more likely to have higher-grade and -stage disease. Langner et al. also noted infrequent HER-2 overexpression and amplification in UTUC patients, which were more likely to occur in high-stage and high-grade tumors (31).

The prognostic value of HER-2 with regard to recurrence and survival in UTUC was debated until larger studies were recently published. In the largest single-center HER-2 study in UTUC patients (32), Sasaki et al. investigated HER-2 status in 171 UTUC patients who underwent RNU and found that overexpression and amplification of HER-2 was associated with early bladder cancer recurrence. These findings were validated by a large multicenter retrospective study of 732 patients (33), in which Soria and colleagues found that HER-2 was overexpressed in 35.6% of patients and linked to pathological characteristics, such as more advanced T stage, presence of lymph node metastasis, high-grade tumor, tumor necrosis, and lympho-vascular invasion. By multivariable analysis, HER-2 overexpression remained associated with disease recurrence and overall and cancer-specific mortality. The authors concluded that HER-2 is a good marker for therapeutic risk stratification and a therapeutic target in certain UTUC tumors.

### Epidermal growth factor receptor

Epidermal growth factor receptor (EGFR), also known as HER-1 or ErbB1, is a transmembrane glycoprotein that has tyrosine kinase activity and is a member of the ErbB family of receptors. The activation of EGFR stimulates its intrinsic intracellular protein tyrosine kinase activity and initiates several signal transduction cascades, leading to DNA synthesis and cell proliferation. Mutations that affect EGFR overexpression or upregulation correlate with several cancers, including UCB (34).

Few studies have determined the influence of EGFR on the prognosis of patients who have been diagnosed with UTUC. In a small single-center study, Tsai et al. detected ErbB1 in 9.5% of UTUC tumors (35). Leibl et al. analyzed the immune-histochemical expression of EGFR in 268 consecutive patients with UTUC who underwent RNU and correlated it with histopathological parameters and patient outcomes (36), finding that EGFR immunoreactivity was present in 55% of cases higher than in previous studies. EGFR staining was associated with higher tumor stage and grade, and its intensity correlated with metastatic disease in the univariate but not multivariate analysis. However, Long et al. recently analyzed 320 UTUC patients and found that EGFR positivity was an independent risk factor for bladder cancer recurrence after RNU (27). Thus, like Ki-67 and HER-2, EGFR expression could be used as a marker of bladder cancer recurrence after radical therapy.

### Nuclear factor-κB (NF-κB)

Nuclear factor-κB (NF-κB) is a complex protein that is expressed in many cells and regulates many genes that control cell proliferation and survival. Typically, NF-κB is inactive and sequestered in the cytoplasm by specific inhibitors under resting conditions. Several stimuli can activate and release NF-κB, allowing it translocate to the nucleus. In its active form, NF-κB regulates the expression of genes that are associated with anti-apoptosis, inflammation, proliferation, invasion, and angiogenesis (37).

A Chinese group studied 90 patients with UTUC by immunohistochemistry (38) and recorded cytoplasmic overexpression of NF-κB in 61% of subjects and nuclear immunoreactivity in nearly 27%. Although no significant association between NF-κB expression and pathological features of aggressive disease was seen, it was an independent predictor of CSS and OS.

### Angiogenesis

Angiogenesis is the process by which new blood vessels form in tissue and is considered a critical event in the initiation and progression of various solid malignancies-without angiogenic activity, solid tumors cannot grow or expand. Several angiogenic factors have been identified in the past several years, including vascular endothelial growth factor (VEGF), platelet-derived growth factor (PDGF), hypoxia-inducible factor 1 (HIF-1), fibroblast growth factor receptor 3 (FGFR 3), vasohibin-1 (VASH1), and thrombospondin-1 (TSP-1). Despite being studied extensively in UCB, there are few reports on angiogenesis-related markers in UTUC.

### Hypoxia-inducible factor 1α (HIF-1α)

Hypoxia-inducible factor 1 protein (HIF-1) is a transcription factor that governs oxygen homeostasis. The levels of the HIF-1α subunit can increase rapidly in response to hypoxia, in turn upregulating factors that are important for tumor expansion. Thus, overexpression of HIF-1α is associated with tumor aggressiveness and prognosis in several malignancies, including UCB (39).

In UTUC, there are few studies on HIF-1α expression and outcomes. Nakanishi et al. performed a retrospective study of 127 patients and found that 55% of samples had immune-histochemical overexpression of HIF-1α (40). This overexpression correlated with high-grade and sessile tumors but not with tumor stage. The authors also observed that HIF-1α overexpression was associated with OS and DFS rates by univariate and multivariate analysis (in all tumors and in invasive tumors), indicating that HIF-1α expression provides information on the prognosis in patients with UTUC.

### Vasohibin-1 (VASH1)

Vasohibin-1 (VASH1) is a protein that is encoded by VASH1 and is an angiogenic molecule that is expressed specifically in endothelial cells and upregulated by VEGF and fibroblast growth factor-2. VASH1 expression is restricted to the endothelial cells of blood vessels in the tumor stroma (41).

Miyazaki et al. were the first group to examine the immune-histochemical expression of VASH1 and microvessel density (MVD) in UTUC with regard to patient outcomes (42). In 171 patients, they noted a significant positive correlation between MVD and VASH1. Elevated levels of VASH1 were significantly associated with higher pathological T stage and high-grade tumors. By multivariate analysis, high VASH1 density was an independent predictor of tumor recurrence and CSS. The authors concluded that although their results are preliminary, VASH1 density is a clinically relevant predictor of patient prognosis in UTUC and could be confirmed as a novel biomarker in external validation studies.

### Thrombospondin-1 (TSP-1)

Thrombospondin-1 (TSP-1) is a potent inhibitor of angiogenesis in various physiological and pathological conditions and suppresses the proliferation of tube-like structures in endothelial cells (43).

Earlier studies reported that TSP-1 expression is inversely related to MVD in bladder cancer patients (44, 45), suggesting that low TSP-1 expression correlates with increased malignant potential, aggressiveness, and poorer outcomes in urothelial carcinomas. There is one paper on TSP-1 expression in UTUC and its impact on the patient’s prognosis. In this retrospective single-center study of 97 UTUC patients who were subjected to RNU (46), the authors analyzed the expression of 4N1K, which is derived from the C-terminal domain of TSP-1 and inhibits angiogenesis in in vivo and in vitro models (47). Miyata and colleagues found that low expression or lack of 4N1K was associated with higher pathological T stage and high-grade tumors. In survival analyses, negativity for 4N1K was a predictor of DFS and CSS in the univariate but not multivariate analysis. Thus, based on these results, the prognostic value of TSP-1/4N1K is limited in UTUC (46).

### Fibroblast growth factor receptor 3 (FGFR3)

Fibroblast growth factor receptor 3 (FGFR3) is part of a tyrosine kinase receptor family that regulates many cellular processes, such as growth, differentiation, and angiogenesis. In general, FGFR3 mutations are associated with low-risk tumors that rarely progress and usually have a good prognosis in bladder cancer patients (48).

In 2009, van Oers et al. reported that FGFR3 mutations occurred at the same frequencies in UTUC and bladder tumors in 280 patients (117 bladder tumors and 163 upper tract tumors) with a median follow-up of 56 months (49). They also claimed that FGFR3 mutations correlated with low-stage tumors and better survival in patients with UTUC and UCB. Burger and colleagues found that FGFR3 mutations were linked to a favorable stage and grade in 172 UTUC patients and benefited the survival rate-24.1% versus 54.2% cancer-related deaths occurred in the mutated group (50). Based on these results, FGFR3 has potential as a marker of low-risk patients and can be used to select candidates for conservative therapies other than RNU, if these findings can be recapitulated in biopsy specimens.

### Cell differentiation

#### Uroplakin

Uroplakins (UPs) comprise a group of four transmembrane proteins (UPs Ia, Ib, II, and III) that are specific differentiation products of urothelial cells and are undetectable in non-urothelial tissues (51). They usually reside at the apical surface and in the cytoplasm of umbrella cells and are absent from intermediate and basal cells. The specificity of UPs to urothelial carcinoma has led to their use as markers of urothelial differentiation.

Ohtsuka et al. measured UPIII by immune-histochemistry in surgical specimens of 71 patients who were diagnosed with UTUC and subjected to RNU (52). In all specimens, there was intense UPIII immunoreactivity in umbrella cells of normal urothelium. In tumor samples, UPIII was positive in 75% of non-muscle invasive tumors and 40% of muscle-invasive disease. UPIII was also expressed in 65% of grade 1-2 and 33% of grade 3 tumors. The CSS of patients who were negative for UPIII was significantly worse than in those who were UPIII-positive, and by multivariate analysis, UPIII expression was a more powerful predictor of CSS than stage and lymph node status. A recent study found that UPII antibody has greater sensitivity than UPIII and is a better marker, even in challenging settings (53).

### Cell adhesion and epithelial-mesenchymal transition (EMT)

The epithelial-to-mesenchymal transition (EMT) is a process in which cells lose their epithelial characteristics and gain the migratory and invasive properties of mesenchymal cells. The EMT is a key step in cancer development and progression. Epithelial cells express high levels of E-cadherin, whereas mesenchymal cells express N-cadherin, fibronectin, and vimentin. Loss of E-cadherin is a fundamental event in the EMT. Downregulation of E-cadherin and increased N-cadherin are hallmarks of EMT in epithelial malignancies and are associated with the acquisition of aggressive phenotypes (54). In normal cells, the cytoplasmic domain of E-cadherin binds to the β or γ subtype of catenin proteins, which in turn are secured to actin microfilaments, thus ensuring cytoskeletal integrity and stable cellular adhesion (55). Loss of cellular adhesion is a critical event in tumor progression, resulting in poorly differentiated and invasive tumors.

### Snail

Snail is an EMT-inducing transcription factor (TF) that represses E-cadherin and thus regulates tumor progression and metastasis. As a result, Snail contributes to the EMT during cell differentiation (8, 56).

Kosaka et al. analyzed 150 patients and demonstrated that nuclear Snail staining was weak in non-muscle-invasive UTUC (56). In contrast, Snail signals were robust in the nuclei of many muscle-invasive tumors. Snail expression was significantly upregulated in high-tumor-stage and high-grade tumors and in tumors with lympho-vascular invasion. By multivariate regression analysis, elevated Snail expression was a significant and independent prognostic predictor of DFS and CSS.

### Cadherins

In many studies of various solid malignancies, patients with low E-cadherin levels were most likely to harbor tumors with features of biologically aggressive disease. This association is consistent with function of E-cadherin as a calcium-dependent glycoprotein that is essential for epithelial tissue integrity. Loss of cellular adhesion results in the detachment of cancerous cells from the primary lesion, promoting invasiveness and metastasis (57). However, the relationship between E-cadherin and outcomes in UTUC is debated.

In 62 UTUC patients, Fromont et al. showed that decreased E-cadherin expression is an independent prognostic factor for DFS and OS (201). In contrast, most subsequent studies of larger cohorts have failed to demonstrate an independent association between E-cadherin expression and disease recurrence (58, 59). Favaretto et al. assessed the clinical significance of lower E-cadherin levels in an international cohort of 678 UTUC patients who were treated with RNU (60), observing that downregulation of E-cadherin in tumor cells correlated with adverse clinicopathological features. They also confirmed that lower E-cadherin expression was linked to a greater probability of disease recurrence and cancer-specific mortality in UTUC. However, when adjusted for the effects of established prognostic factors by multivariable analysis, E-cadherin expression lost its independent prognostic value and thus might have limited use in clinical practice. Nevertheless, it can be used to identify more aggressive tumors in the preoperative setting to indicate perioperative chemotherapy and extended lymph node dissection, in addition to RNU, if these results can be validated in biopsy specimens.

Muramaki et al. examined N-cadherin in UTUC in 59 patients, reporting that its expression was an independent prognostic factor of intra- and extravesical recurrence after RNUl (59) but, they did not analyze any predictors of CSS in this cohort. More recently, Abufaraj et al. measured N-cadherin expression in a multi-institution study of over 600 patients (61). N-cadherin positivity was seen in 43% of patients and was associated with features of aggressive disease (advanced tumor stage, lymph node metastases, and sessile architecture) and disease recurrence but not CSS or OS in the univariate analysis. Similarly, after adjustments for the effects of confounders, N-cadherin was not associated with any survival outcome.

### Catenins

Catenins are a family of proteins that exist in complexes with cadherins. The primary mechanical function of catenins is to connect cadherins to actin filaments, specifically in the adhesion junctions of epithelial cells, ensuring cytoskeletal integrity and stable cellular adhesion (56, 62). The catenins that were identified in human cancers became known as α-catenin, β-catenin, and γ-catenin.

Typically, studies determined the prognostic value of catenins in urothelial carcinoma with cadherins as part of a combined panel of EMT markers (55, 63). In UCB, Clairotte et al. analyzed 101 patients with bladder cancer (71 T1 and 30 T2/T3 tumors) and observed a highly significant correlation between decreased expression of all catenins and higher TNM stage (55). By multivariate analysis, only γ-catenin was an independent predictor of DFS in patients with stage T1 bladder urothelial tumors.

In UTUC, few studies have examined the expression of catenins as prognostic markers. Izquierdo et al. measured α, β, and γ-catenin levels in 70 tumors samples from UTUC patients by immunohistochemistry and correlated them with outcomes- (64) in the multivariate analysis, abnormal β-catenin expression was an independent prognostic factor of tumor progression and CSS. In a cohort of 20 patients, Reis et al. reported that moderate expression of γ-catenin was associated with a lower DFS rate, albeit insignificantly (63); there was no relationship between α-catenin and β-catenin expression and tumor recurrence. Also, the authors found that downregulation of α-catenin was more frequent in larger tumors. In conclusion, the data on catenins as prognostic markers in UTUC remain weak; thus, none of them can be used for clinical decision-making.

### Metalloproteinases

Matrix metalloproteinases (MMPs) are part of a large family of proteases that degrade many types of extracellular matrix proteins. Tumor cell invasion and metastasis depend on the proteolytic destruction of surrounding matrix components. Thus, many studies have determined the effects of MMPs on cancer invasion and progression (65-67).

In UTUC, Nakanishi et al. analyzed MMP-2 expression and correlated it with outcomes (68), noting a relationship between MMP-2 levels and tumor stage. Also, in the univariate analysis, MMP-2 expression was associated with OS but did not reach statistical significance in the multivariate analysis. Subsequently, Miyata et al. measure MMP-2 and MMP-9 in 91 patients who underwent RNU (69), reporting that they correlated with higher pT stage but that they were not independent predictors of CSS. In 2005, Kamijima et al. studied 69 UTUC patients and did not observe any link between MMP-2 and MMP-9 expression and high-risk disease (70).

Conversely, Li et al. demonstrated that high MMP-11 expression in 340 UTUC patients and 295UCB subjects was significantly associated with advanced pT status, nodal metastasis, high histological grade, vascular and perineural invasion, and frequent mitoses (71). In the multivariate analysis, MMP-11 was independently associated with CSS and the development of metastasis. Based on the most recent literature, MMP-2 and MMP-9 have limited value as predictive markers in UTUC. In contrast, MMP-11 has shown promising results in a large multicenter retrospective study and is thus a potential novel prognostic and therapeutic target.

### Apoptosis

Apoptosis is the highly regulated and controlled process of programmed cell death. It can be initiated by an intrinsic or extrinsic pathway, both of which induce cell death by activating proteases, called caspases. Inhibition of apoptosis and apoptotic pathways can result in tumorigenesis and cancer progression, because it allows tumor cells to survive longer and resist harmful stressors (72). Several markers of apoptosis have been identified and studied in several malignancies, including survivin, caspase-3, FAS (first apoptosis signal), bcl-2, and bax. Some of these molecules have been examined in UTUC and are summarized below.

### Survivin

Survivin is a member of a family of proteins that inhibits apoptosis by blocking downstream caspase activity (73). In 2009, a South Korean group analyzed the expression of several apoptosis-related markers in UTUC and their association with clinical and pathological features, disease recurrence, and survival (74). They found that 20% of patients had altered expression of survivin. Although survivin expression did not correlated with any clinical or pathological characteristics, in the multivariate analysis, it was the only marker that was independently associated with disease recurrence and survival. Yet, well-designed studies with larger cohorts have been unable to confirm these findings (75, 76, 21).

A recent large multicenter study by Mathieu et al. in 732 patients who were diagnosed with UTUC analyzed survivin expression (75), finding that nearly 40% of tumors had altered survivin levels, which were associated with more advanced pathological tumor stage, lymph node metastasis, lympho-vascular invasion, tumor necrosis, and tumor architecture. In the univariate analysis, altered survivin correlated significantly with DFS and CSS but did not achieve independent predictive status by multivariate analysis.

### Bcl-2 and caspase-3

Bcl-2 is an antiapoptotic protein that blocks caspase activation by inhibiting the mitochondrial release of cytochrome-c (77). In UCB, studies on bcl-2 and caspase-3 are conflicting. Recent studies have shown that overexpression of bcl-2 and loss of caspase-3 are associated with higher pathological stage, disease recurrence, and cancer-specific mortality rates (78). Jeong et al. analyzed several apoptosis-related markers in 112 consecutive UTUC patients (74), reporting that 27% and 24% of patients had altered expression of bcl-2 and caspase-3, respectively-the latter of which correlated with pathological tumor stage and grade; bcl-2 was not linked to any clinico-pathological parameter. In the survival analysis, altered caspase-3 expression was significantly associated with an increased probability of disease recurrence and CSS. When adjusted for the effects of pathological features by multivariate analysis, caspase-3 did not reach statistical significance. bcl-2 overexpression was not associated with disease recurrence or survival in the univariate or multivariate analysis.

In 2013, Yoshimine et al. published data on the expression of bcl-xl, a bcl-2 family protein, from 175 patients with UTUC (79). They also tested a specific inhibitor of bcl-xl, bafilomycin A1 (BMA), in in vitro and xenograft mouse models. This group observed that patients with high bcl-xl levels had a significantly lower 5-year CSS rate (53%) than those with low expression (77%). By multivariate analysis, high bcl-xl expression was an independent prognostic factor for CSS. Further, BMA inhibited urothelial cell proliferation in vitro by inducing apoptosis and suppressed tumor growth in mouse models. The authors suggested that in addition to being a good prognostic marker in UTUC patients, bcl-xl is a promising therapeutic target.

## CONCLUSIONS

UTUC is a highly heterogeneous disease for which there is a significant lack of clinical predictive factors for accurately stratifying patients with regard to treatment decisions. Pathological predictive factors might be effective in determining the risk of aggressive disease, but information on them becomes available only after definitive therapy, precluding patients from receiving more efficacious preoperative chemotherapy or extended lymph node dissection. Recent molecular studies have examined the various pathways in tumor behavior and carcinogenesis, and the incorporation of tissue-based markers into prognostic tools might help identify patients who could benefit from intensified therapy and monitoring.

In this review, we have described the existing data on tissue-based markers in UTUC (Box 1), their prognostic impact, and how they might affect clinical decision-making in this disease. Despite the growing body of evidence on tissue-based markers in UTUC in the past several decades, none of these biomarkers is used in daily practice. The number of studies on tissue-based markers has risen substantially, but many discrepancies in their results remain. Most such reports have been based on single centers and small cohorts, with a low level of evidence. Few studies have had their findings validated, and the incorporation of tissue-based markers into prognostic models have been unable to improve their accuracy significantly (28).

BOX 1 **Molecular prognostic markers in UTUC**

- p53 overexpression (inactivation/accumulation)
- Cyclins A and E overexpression
- Loss or lower expression of p21 and p27
- Overexpression of Ki67 (proliferation index)
- HER-2 overexpression and amplification
- EGFR overexpression
- NF-κB overexpression
- HIF-1α overexpression
- High levels and high density of VASH1
- Loss or lower expression of TSP-1
- FGFR mutation/overexpression (protective)
- Negative UPII and UPIII expression
- Snail overexpression
- Loss of E-cadherin expression
- Positive N-cadherin expression
- Abnormal expression of α, β and γ-catenin
- Overexpression of MMP-2, MMP-9 and MMP-11
- Altered survivin expression
- Altered expression of Bcl-2 and caspase-3 (apoptosis related markers)

Another problem in evaluating tissue-based markers in UTUC is the heterogeneity of the cohorts that are usually recruited. In a review, Netto et al. described the two pathogenetic pathways in UCB (80). One pathway was associated with non-muscle-invasive bladder cancer and comprised disruption to PI3K-AKT-mTOR signaling and alterations in FGFR3 and the oncogene HRAS. Conversely, the primary genetic alterations that underlie muscle-invasive bladder cancer involve tumor suppressor genes that encode proteins that regulate the cell cycle and apoptosis, including TP53 and RB1. UCB studies typically include patients with non-muscle-invasive disease who have undergone transurethral resection or received intravesical therapies or patients with muscle-invasive disease who have been subjected to radical cystectomy. In contrast, studies on UTUC generally comprise patients with any stage or grade who have undergone RNU, thus including too many disparate pathogenetic pathways in the same cohort.

However, certain biomarkers appear to be promising tools in UTUC. In large multi-institution retrospective studies, Ki-67, HER-2, EGFR, and N-cadherin were independent prognostic factors for bladder cancer recurrence after RNU and could be used as markers for intravesical therapies and bladder surveillance. Most of the tissue-based markers that have been studied correlate with high-grade and high-stage disease-one exception is FGFR3, which is linked to low-risk tumors. Other biomarkers have had good results as independent prognostic factors for survival (p53, Ki-67, HER-2, and MMP 11).

These studies have been performed in RNU specimens, allowing us to stratify patients for adjuvant chemotherapy and intravesical therapy or intensify surveillance. However, the preoperative identification of patients with aggressive versus low-risk disease with regard to indications for neoadjuvant chemotherapy and extended lymph node dissection compared with conservative therapy remains a critical issue. Thus, the validation of these biomarkers in biopsy specimens would be invaluable for treatment decision-making.

Recently, immunological check point markers, such as PD1/PDL-1, CD28, CTLA-4, and T-cell receptors and proteins (e.g., FOXP3) have been evaluated as prognostic factors and targets for new drugs in several solid tumors, including UCB (81). We hope that these molecules will also be investigated as prognostic factors in UTUC.

In conclusion, tissue-based markers have the potential to improve the clinical prediction of outcomes in UTUC, necessitating their further development. Prospective studies that validate the function of these markers are being awaited, for which multi-institution collaborations are necessary. An innovative panel of biomarkers that encompass a role pathway or several pathways might valuable, due to the dynamic processes of tumorigenesis, invasion, and progression, and creating accurate models to predict patient outcomes and the response to therapy would be a significant advance toward improving survival.
